# Enabling technologies and green processes in cyclodextrin chemistry

**DOI:** 10.3762/bjoc.12.30

**Published:** 2016-02-15

**Authors:** Giancarlo Cravotto, Marina Caporaso, Laszlo Jicsinszky, Katia Martina

**Affiliations:** 1 Dipartimento di Scienza e Tecnologia del Farmaco and NIS - Centre for Nanostructured Interfaces and Surfaces, University of Turin, Via P. Giuria 9, 10125 Turin, Italy

**Keywords:** ball milling, cyclodextrin, microwaves, synthesis, ultrasound

## Abstract

The design of efficient synthetic green strategies for the selective modification of cyclodextrins (CDs) is still a challenging task. Outstanding results have been achieved in recent years by means of so-called enabling technologies, such as microwaves, ultrasound and ball mills, that have become irreplaceable tools in the synthesis of CD derivatives. Several examples of sonochemical selective modification of native α-, β- and γ-CDs have been reported including heterogeneous phase Pd- and Cu-catalysed hydrogenations and couplings. Microwave irradiation has emerged as the technique of choice for the production of highly substituted CD derivatives, CD grafted materials and polymers. Mechanochemical methods have successfully furnished greener, solvent-free syntheses and efficient complexation, while flow microreactors may well improve the repeatability and optimization of critical synthetic protocols.

## Review

**Figure 1:**
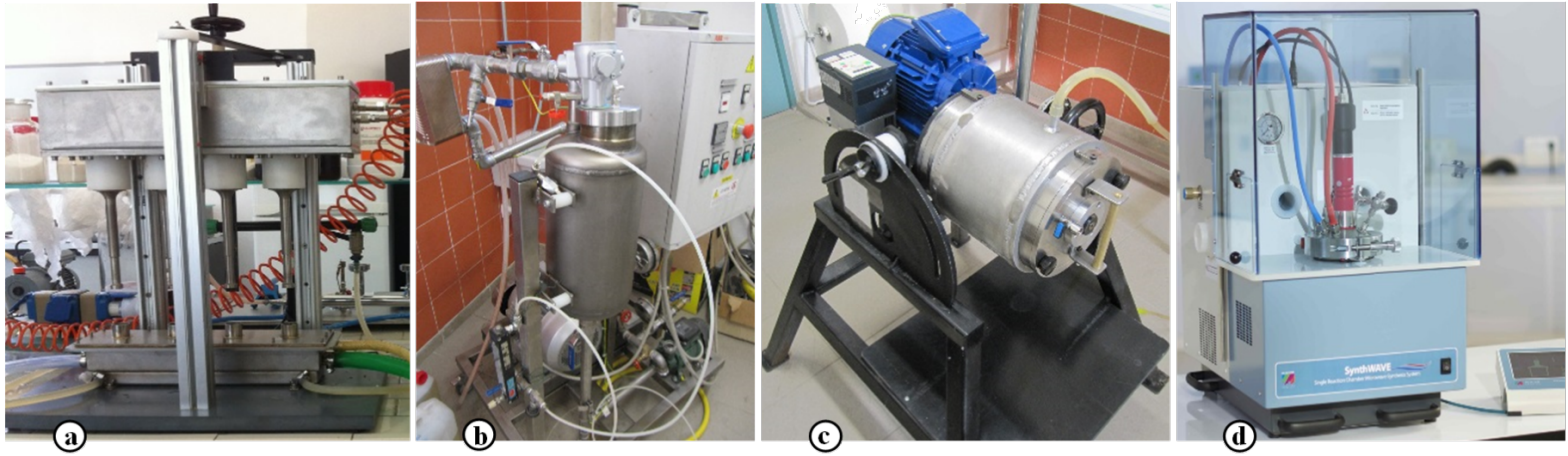
(a) Multihorn-flow US reactor, (b) Cavitational turbine, (c) Pilot-scale BM, (d) High-pressure MW reactor.

The last decade has witnessed the development of highly efficient alternative synthetic methods which make use of new enabling technologies. The need for a more rational approach to the synthesis of cyclodextrin (CD) derivatives has led to several energy sources been tested for their ability to activate C–C and C–X bond formation. In recent years non-conventional energy sources, such as microwaves (MW), ultrasound (US), ball mills (BM) and microreactors have made access to CD derivatives much simpler, as have heterogeneous catalysts and greener solvents. Besides batch reactors, in the last decade these techniques have been adapted to flow systems, which provide greater efficiency, flexibility and lower energy consumption, or in high-throughput applications. Our experience in process intensification and innovative reactors took advantage from flow-multihorn US systems (Figure 1a) and cavitational turbines (Figure 1b) to optimize mass transfer via intense cavitation [1–2]. Similarly, we have accumulated experiences with mechanochemical conditions that open the way to solventless reactions even on a pilot scale (Figure 1c) [3]. The latest generation of dedicated MW reactors, which enable operators to quickly screen reaction conditions by means of parallel tests across a wide range of operative conditions, has provided outstanding MW-assisted synthesis results (Figure 1d) [4]. While most researchers will most likely be acquainted with the potential of dielectric heating, the specific conditions needed to let react CDs efficiently and selectively are often overlooked.

**Figure 2:**
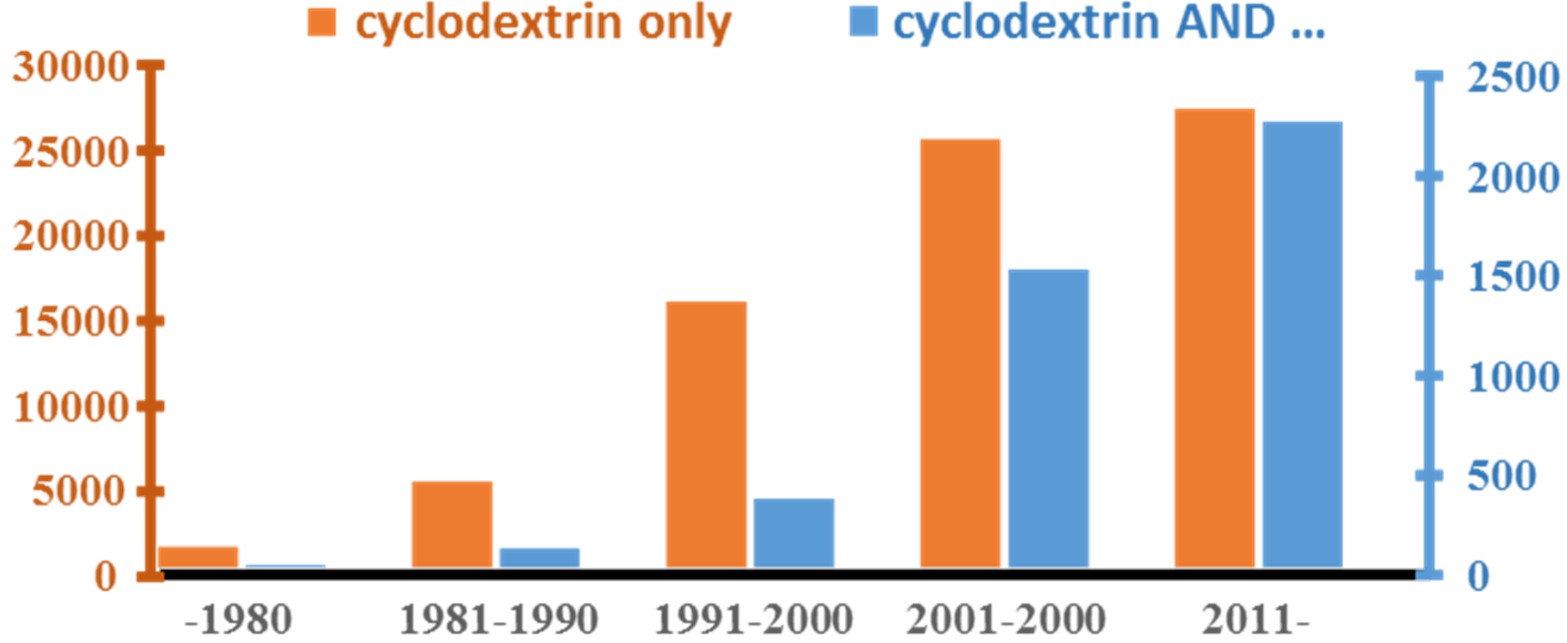
Trends in CD papers and CD use in green chemical processes.

The current trends on CDs’ literature and their application in green protocols are clearly depicted in Figure 2. The present literature survey with identical keyword combinations has been done in two major databases [5–6]. The results were partially overlapped only in the full text searches and approximately 4000 records have been found. Further reduction of records, less than 2500, by searching in Title/Abstract/Keyword fields only resulted in more relevant publications. Only 10% roughly of the recently published papers on CDs are dealing with sustainable technologies and only few works are comparing data with conventional synthetic protocols.

Mechanochemical syntheses are typically carried out in BM and also in low-frequency US reactors [7]. This technique has recently developed into a genuine eco-friendly alternative when manufacturing inorganic, organic and metal-organic compounds as well as supramolecular composites, which may differ to those prepared via conventional routes [8]. Higher versatility and selectivity offer a wide range of applications and may facilitate the purification steps [9]. Noteworthy examples are the mechanochemical derivatization of saccharides [10–11], the functionalization of CDs and their complexation with organic molecules [12]. Solid state organic reactions using CD cavities as nanoreactors have also been reported [13].

**Figure 3:**
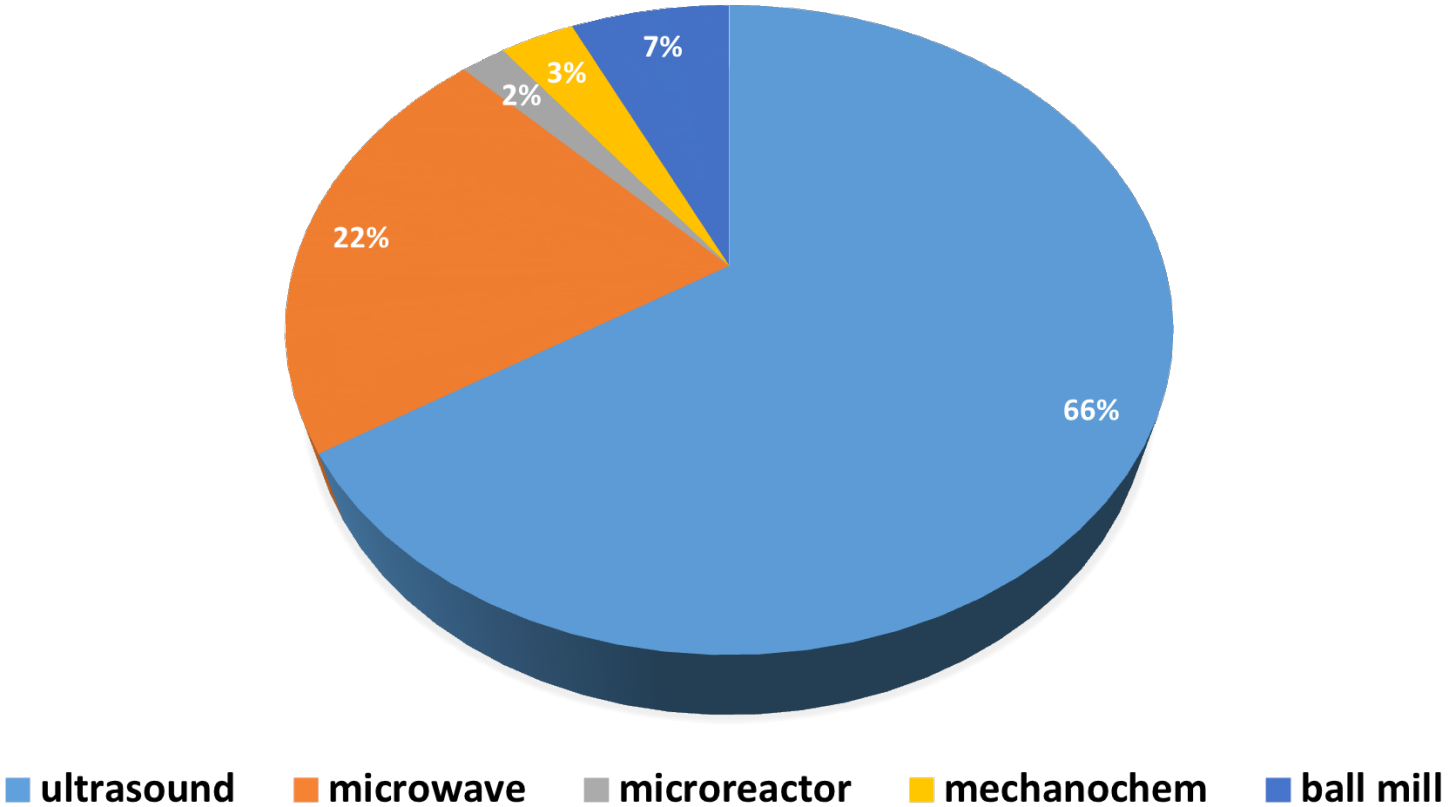
Distribution of energy efficient methods in CD publications.

Among non-conventional techniques, the largest number of papers is dealing with US-assisted CD solubilization or re-dissolution and in a minor extends CD derivatization. Analogously, ball milling is mostly used in the preparation of CD complexes rather than synthetic preparations. MW-assisted CD chemistry covers 1/4–1/5 of the whole literature as seen in Figure 3, mainly focused on synthetic applications.

**Figure 4:**
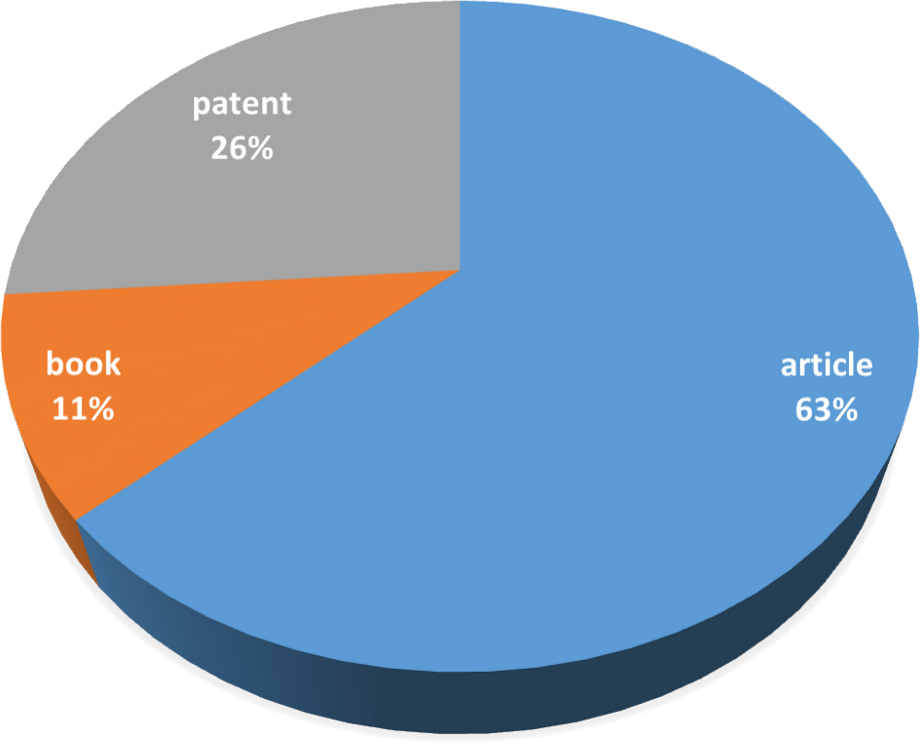
Document type dealing with CD chemistry under non-conventional techniques (conference proceedings and dissertations are handled as books).

As seen in Figure 4, the cake of document types dealing with CD chemistry under non-conventional techniques shows a similar distribution as observed in general CD publications, namely 70% article, 20% patents and 10% books (including non-journal conference proceedings and dissertations).

**Figure 5:**
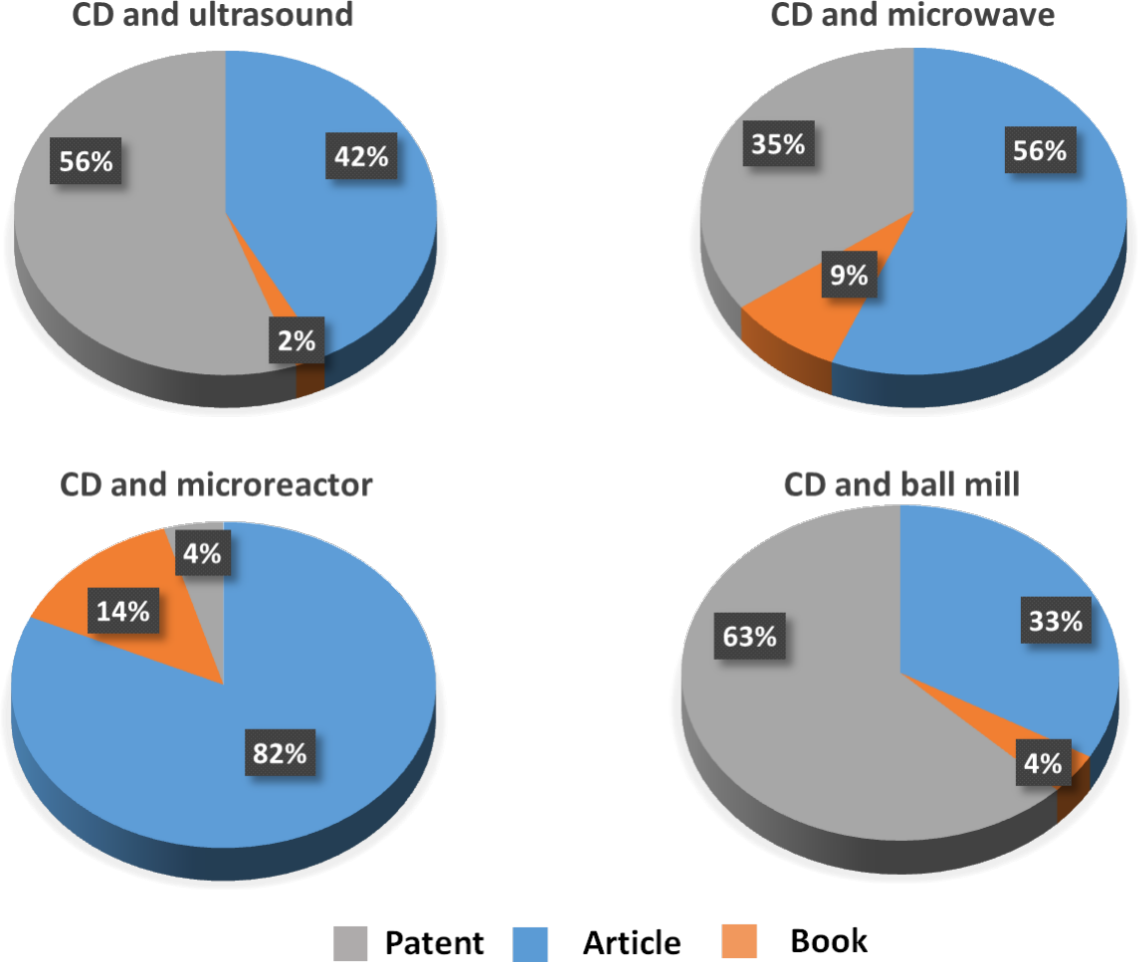
Document type dealing with sustainable technologies in CD publications.

However, industrial applications of such enabling techniques are a priori restricted to US and BM, owing to safety concerns on big scale MW reactors (Figure 5). Microreactors are a relatively new technologies and the small number of patents may also derive from solubility limitation.

This review highlights the most recent synthetic advances in CDs’ chemical modification and some perspectives that make use of non-conventional methods and energy sources. Reaction times and yields have been compared with classic procedures to highlight the huge advantages and potential scalability of these so-called enabling technologies that maximize heat and mass transfer.

Although many advances have been made during the past decade, the most exciting results in this field are surely yet to come.

### Ultrasound

US irradiation is an environmentally friendly technique that is well suited to the selective chemical modification of CDs from native α-, β- and γ-CD. The use of this method in heterogeneous phase reactions, such as reductions and “click reactions” [14], is well known, as is its use in full CD derivatization in combination with MW irradiation.

#### Monosubstituted CD derivative preparation

Mono 6^I^-(*p*-toluenesulfonyl)-β-CD is the most popular of the CD derivatives because it is a key intermediate in the synthesis of important amino, azido, thio, thiocyanate and halo-derivatives. 6^I^-(*p*-toluenesulfonyl)-β-CD was efficiently prepared in an US-assisted procedure by reacting β-CD with tosyl imidazole (TsIm) [15]. Great advantages, in terms of yields, reaction times and product purity, were obtained by using a cavitating tube (40 min, 19.2 kHz, 20 W, yield: 55%).

**Scheme 1:**
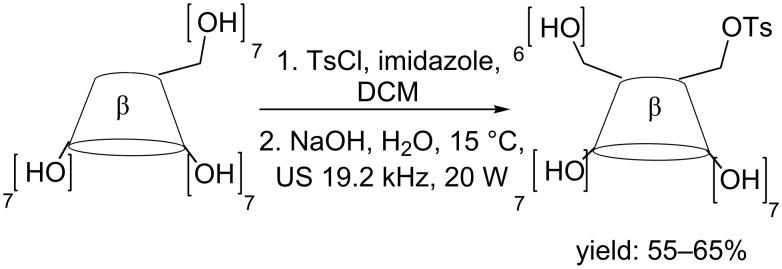
Synthesis of 6^I^-(*p*-toluenesulfonyl)-β-CD.

Thanks to the fast US-assisted inclusion complex formation between β-CD and TsIm reaction times have been dramatically reduced (10 min vs 1–2 hours, Scheme 1).

More recently, Zheng et al. have described the synthesis of this important intermediate via an US-assisted method in basic water solution [16].

The synthesis of 6^I^-amino-6^I^-deoxy-β-CD was also improved by using non-conventional techniques. The catalytic hydrogenation of 6^I^-azido-6^I^-deoxy-β-CD using Pd/C was achieved under US irradiation in MeOH/H_2_O in 20 min (20.4 kHz, 80 W, yield: 88%); hydrogen was supplied at 1 bar pressure [15].

**Scheme 2:**
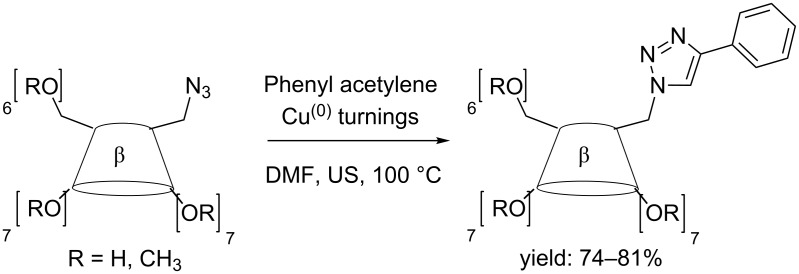
Example of CuAAC with 6^I^-azido-6^I^-deoxy-β-CD and phenylacetylene.

Sonochemical metals depassivation in organometallic reactions is well established [17]. A typical example is the Cu(0)-catalysed azide–alkyne cycloaddition (CuAAC) that can be further enhanced by simultaneous US/MW irradiation [18]. The formation of triazole-substituted CDs has been investigated by US irradiation and products can be synthesized in 2–4 hours (Scheme 2) [19].

**Scheme 3:**
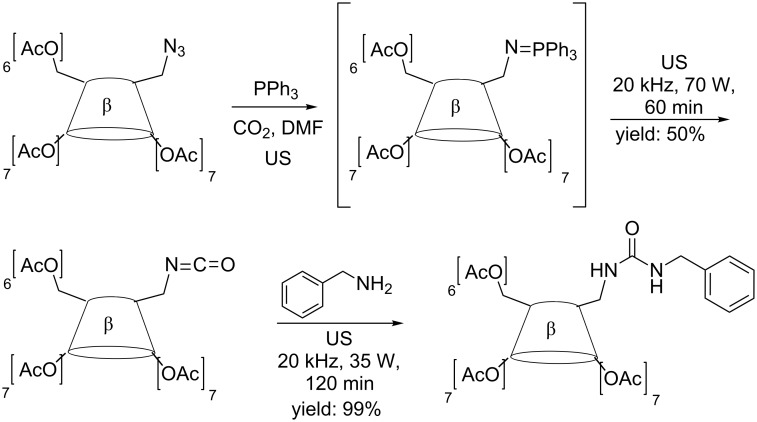
Synthesis of 6^I^-benzylureido-6^I^-deoxy-per-*O*-acetyl-β-CD.

Scondo et al. have reported a preliminary study on sonochemical Staudinger-aza-Wittig tandem reactions [20] proving that isocyanate and urea formation is strongly favored. However, the applied power must be optimised for the best conversions of azido-CD into urea to be obtained and if lower efficiency in the second step is to be avoided. 6^I^-Benzylureido-6^I^-deoxy-per-*O*-acetyl-β-CD was obtained in shorter reaction times and excellent yields as compared to conventional conditions (Scheme 3).

**Scheme 4:**
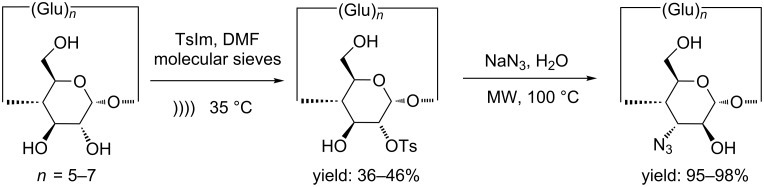
Synthesis of 3^I^-azido-3^I^-deoxy-*altro*-α, β- and γ-CD.

Tosylation of the secondary rim of the CD can be efficiently carried out under US irradiation. This efficient regioselective modification is performed in the presence of tosyl imidazole and molecular sieves under US irradiation. As shown in Table 1, the reaction time was shortened to 2 h for α-CD (yield: 36%), 1 h for β-CD (yield: 40%) and 45 min for γ-CD (yield: 46%) (Scheme 4) [21].

**Table 1:** Selected examples of conventional and non-conventional preparation of monosubstituted CDs.

Product	Reaction conditions	Time	Yield (%)

6^I^-tosyl-β-CD	β-CD, Tosyl chloride, NaOH, water, rt [22]	18 h	34
6^I^-tosyl-β-CD	β-CD, TsIm, NaOH, water, US, 20 W [15]	30 min	55–60
2^I^-tosyl-α-CD	α-CD, TsIm, DMF, mol sieves, rt [23]	50 h	35
2^I^-tosyl-α-CD	α-CD, TsIm, DMF, mol sieves, US 20 W [21]	2 h	36
2^I^-tosyl-β-CD	β-CD, TsIm, DMF, mol sieves, rt [23]	50 h	36
2^I^-tosyl-β-CD	β-CD, TsIm, DMF, mol sieves, US 20 W [21]	1 h	40
2^I^-tosyl-γ-CD	γ-CD, TsIm, DMF, mol sieves, rt [24]	120 h	36
2^I^-tosyl-γ-CD	γ-CD, TsIm, DMF, mol sieves, US 20 W [21]	45 min	46
6^I^-amino-β-CD	6^I^-azido-6^I^-deoxy-β-CD, Pd/C, N_2_H_4_, MeOH, H_2_O, reflux [22]	20 min	90
6^I^-amino-β-CD	6^I^-azido-6^I^-deoxy-β-CD, Pd/C, H_2,_ MeOH, H_2_O, US 20 W [15]	2 h	88

In Table 1 we compared the preparation of several monosubstituted CDs under conventional condition or under US irradiation. The data show that reaction time were dramatically reduced and the yield was generally slightly increased. Under US irradiation, the 6^I^-amino-β-CD was obtained by catalytic hydrogenation, while under conventional conditions the reduction of azido β-CD was obtained by a Staudinger reaction or in the presence of hydrazine.

A new generation of organophosphate scavengers has been obtained by Le Provost et al. [25] in which β-CD was regioselectively monosubstituted at O-2 using a bromomethyl pyridine derivative under US irradiation to avoid polysubstitution.

#### Preparation of persubstituted CD derivatives

The complete substitution of all hydroxy groups is difficult because steric hindrance increases upon substitution, the secondary face may be attacked before the last primary hydroxy group has completed the reaction.

Totally persubstituted products are usually obtained in low yields, whereas significant increases in yields have been achieved in reduced reaction times thanks to our sonochemical protocol (35 kHz bath at 20 °C, 160 W; 20 kHz cooled horn, −20 °C, 600 W). We prepared a series of *O*-peralkylated β- and γ-CDs which are commonly used as stationary phases in high-resolution gas chromatography or as drug carriers [26].

CDs and their persubstituted derivatives have recently received a great deal of attention from the field of chromatographic separations. The development of new CD derivatives as important selectors for analytical chiral recognition have been performed [27]. We prepared heptakis(6-O-TBDMS-2,3-O-methyl)-β-CDs with a second CD unit in the 2 position or a (*R*)-Mosher acid moiety [28].

#### Preparation of second generation CD derivatives: dimers, and CD hybrids

Bis-CDs and their metal complexes have been extensively studied as versatile receptors for molecular recognition and building blocks for functional materials.

Due to the binding of two adjacent CD units, bridged bis-CDs display high binding abilities and molecular selectivities compared to native and monosubstituted CDs. A well-organized pseudo-cavity may be provided by the linker that in turn offers additional binding interactions with guest molecules.

**Scheme 5:**
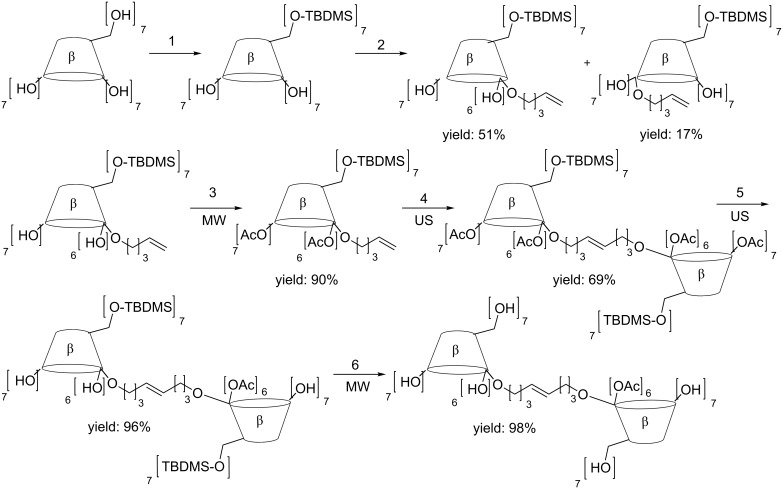
Synthesis of 2-2’ bridged bis(β-CDs). Reaction conditions: 1) TBDMSCl, imidazole, dry pyridine, stirring rt, 8 h; 2) 5-bromopentane, LiH, dry THF–DMSO, reflux, 4 h; 3) acetic anhydride, dry pyridine, MW, 50 °C, 1 h; 4) Grubbs’ catalyst, Ar, dry CH_2_Cl_2_, US, 34 °C; 5) KOH, 2 M, MeOH, H_2_O; US; 40 °C, 30 min; 6) AcCl 2% in MeOH; CH_2_Cl_2_, MW, reflux, 15 min.

New sonochemical protocols for the preparation of bis(β-CDs) bearing 2-2′ and 3-3′ bridges as new carriers for gadolinium complexes have been reported (Scheme 5) [29]. These new CD dimers were promising candidates for MRI applications because their Gd(III)-adducts endowed with high relaxivities thanks to much larger molecular masses than the contrast agents themselves.

Furthermore, the potential use of cyanine/β-CD carrier systems has been evaluated via in vitro experiments on HeLa cells and the monitoring of cell entrance via confocal laser scanning microscopy [30]. Several types of dye moiety/CD derivatives have been suggested as "switch on" or "switch off" fluorescent chemical sensors. In these systems, the complexation with a guest molecule allows to enhance or decrease the fluorescence intensity. Two water-soluble cyanine/β-CD derivatives have been efficiently prepared via CuAAC under simultaneous US/MW irradiation at 75 °C for 2 h (MW 15 W and US 20 W) in good yields (23% and 33%). These dyes were used as versatile carriers for drug delivery and optical imaging.

#### Preparation of CD-grafted materials and CD-based polymers

The reaction of β-CD with diphenyl carbonate (DPC) or hexamethylene diisocyanate (HDI) afforded crosslinked, insoluble polymers. We synthesized these systems and tested as sequestering agents for naringin [31]. These syntheses were carried out under US with shorter reaction times and smaller particle size distribution.

**Scheme 6:**
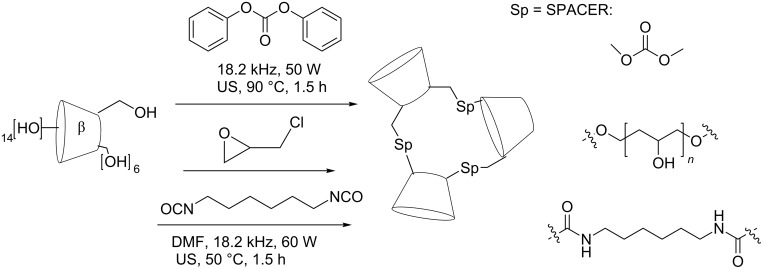
Insoluble reticulated CD polymer.

To investigate the cosmeto-textile applications of CD-grafted materials, a new fabric based on β-CD-grafted viscose loaded with aescin formulations was prepared. This material was designed for the treatment of venous and lymphatic legs. An efficient US-assisted synthetic procedure to graft viscose using a diisocyanate cross-linker was reported (Scheme 6) [32].

**Scheme 7:**
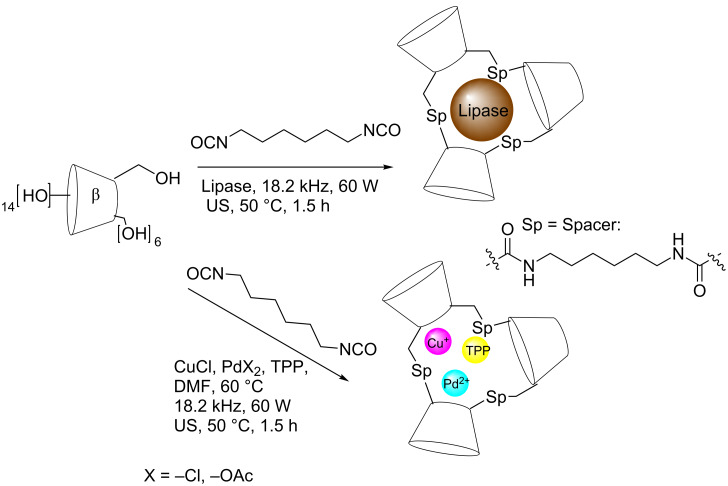
CD-HDI cross linked polymers.

Sonochemical reticulation with HDI was used in the preparation of a new series of solid cross-linked α-, β- and γ-CD-based catalysts containing Cu(I) or Pd(II) [33]. Sonication breaks up intermicellar interaction and may promote the formation of metal nanoparticle clustering. Cu(I)-based system have been used in alkyne/azide [3 + 2] cycloadditions, while Pd(II)-based catalysts have been used in C–C couplings reactions (Scheme 7) [34].

An example of water-soluble β- and γ-CD/chitosan derivatives have been studied for binding Gd(III) chelates that bear hydrophobic substituents and negative charges [35]. These bio-polymers were easily prepared in two reaction steps by reacting CDs with maleic anhydride followed by activation with carbodiimide to form amide linkages with amino groups of chitosan. The esterification of CD was promoted by MW irradiation, while the chitosan coupling used a water-soluble carbodiimide, *N*-(3-dimethylaminopropyl)-*N*-ethylcarbodiimide hydrochloride, under US.

A mild sonication at rt using HDI enabled efficient CDs reticulation in the presence of lipases (Scheme 7) whose biocatalytic activity was preserved in the final solid cross-linked β-CD enzyme [36].

Nanosponges are nanostructured materials made of hyper-cross-linked CDs [37]. The capacity of these materials to encapsulate a great variety of substances could be used to design innovative drug carriers, to protect degradable substances and to improve the aqueous solubility of poorly water-soluble molecules. α-, β- and γ-CDs were reacted solventless with diphenyl carbonate or carbonyldiimidazole under US (up to 90 °C). These nanosponges may resolve some active ingredients drawbacks, such as instability, degradation, poor solubility and toxicity, while they can also be used as carriers for inhalation and oral administration treatments [38].

New hybrid materials have been created from a combination of carbon nanotubes (CNTs) and β-CD [39] affording a peculiar cost-effective fibre. Functionalized β-CD was covalently linked to CNTs and this derivative was immobilized into the wall pores of the hollow fibre under US [40].

### Microwaves

A number of general books and reviews discuss in detail the state-of-the-art of MW-assisted organic synthesis and tailor-made MW reactors have been developed for green organic synthesis [41–42]. The most recent generation of professional reactors dramatically increased the applications of MW-assisted organic synthesis thanks to a high power density (up to 1.5 kW/L), high temperature (up to 300 °C) and pressure (up to 200 bar) together with multi-gas inlets. Considering that MW ovens can be interfaced with autosamplers and that new MW reactors can accommodate multiple racks, this technique is well suited for fast optimization of organic protocols and parallel synthesis. The most impressive advantage of the MW technology is the appearance of the kilolab-scale reactors and their special versions that are operating in continuous flow mode [4,43–44].

#### Preparation of monosubstituted CD derivatives

**Scheme 8:**
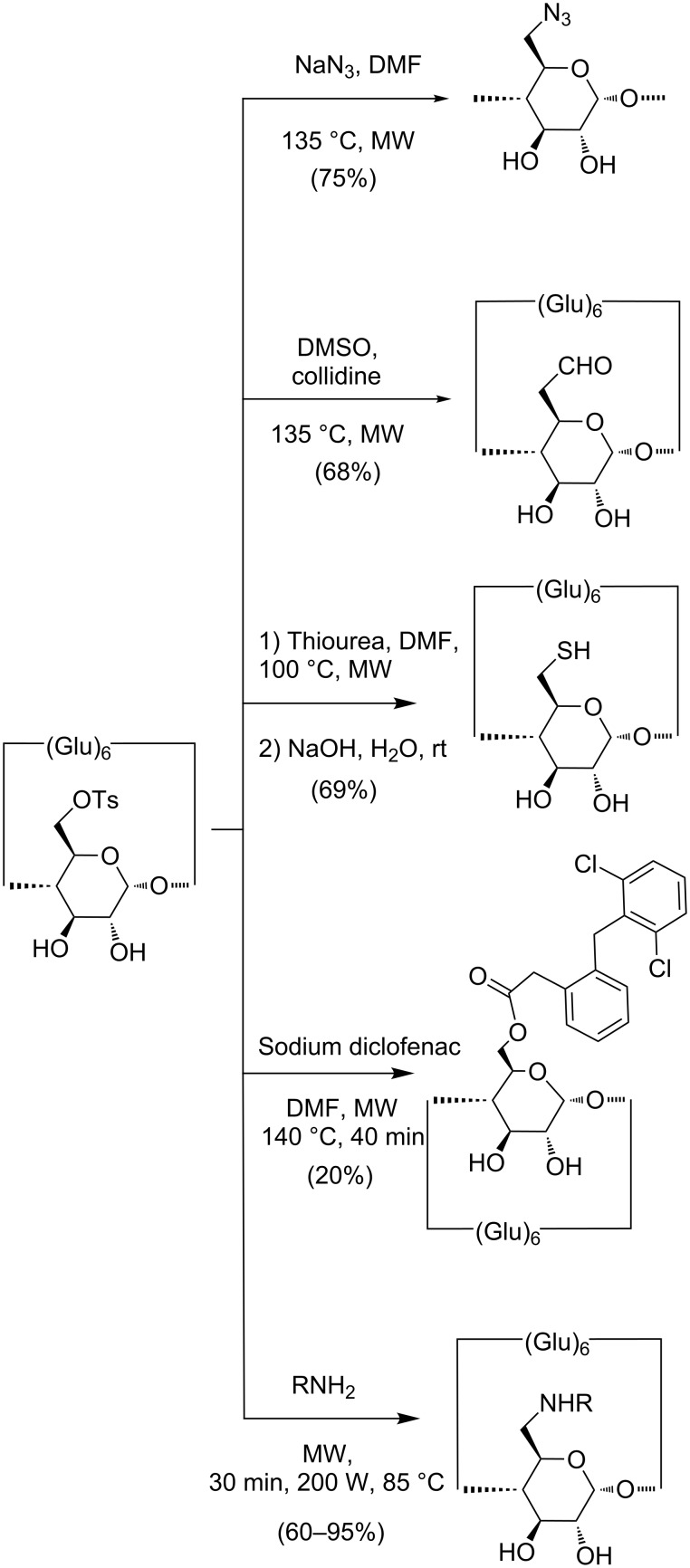
Derivatization of 6^I^-(*p*-toluenesulfonyl)-β-CD by tosyl displacement.

MW irradiation has been exploited in the synthesis of mono and persubstituted CDs. Several syntheses of CD derivatives have been successfully carried out under MW with higher yield, higher purity, and short reaction time. While US irradiation has found use in the optimization of synthetic protocols for the preparation of versatile intermediates, such 6^I^-(*p*-toluensulfonyl)-β-CD from native β-CD, MW irradiation has proved to be extremely efficient in further derivatization, such as the nucleophilic substitution of monohalogenated and monotosylated CDs (Scheme 8) .

The 6^I^-azido-6^I^-deoxy-β-CD, an extremely versatile intermediate, has been obtained from the displacement of the tosylate group under MW. The reaction time was cut from several hours to 2 min (200 W max, 85 °C) and the formation of side products was reduced [15]. 6^I^-(*p*-Toluenesulfonyl)-β-CD was converted to 6^I^-formyl-β-CD via DMSO oxidation in MW with collidine in 15 min (110 W, 135 °C). MW irradiation promoted the syntheses of 6^I^-deoxy-6^I^-thio-β-CD and 6^I^,6^IV^-dideoxy-6^I^,6^IV^-dithio-β-CD via nucleophilic substitution of the primary tosylate ester in C-6 with thiourea followed by basic hydrolysis. The reaction gave the thiouronium salt after 1 h of irradiation at 100 °C while 20 h heating at 90 °C are required under conventional conditions [21].

While the previous experiments were performed in a multimode MW oven (MicroSynth-Milestone, Italy), a similar approach was used for the preparation of an ester prodrug of diclofenac and β-CD, but in a monomode MW oven (CEM Discover S-class MW reactor). The reaction was heated at 140 °C for 40 min and the diclofenac β-CD derivative was obtained with a yield of 20% [45]. Analogously, a general MW-assisted procedure for the synthesis of 6^I^-amino-6^I^-deoxy-β-CD has been reported by Puglisi et al. The reactions were performed in a MW oven (CEM Explorer) for 30 min at 200 W and 85 °C [46].

In the Table 2we compared MW vs conventional procedures in the preparation of several monosubstituted derivatives. Besides a slight improvement of formyl and thio derivative yield, the data show a significant reaction rate acceleration.

**Table 2:** Selected examples of conventional and MW-assisted preparation of monosubstituted CDs.

Product	Reaction condition	Time	Yield (%)

6^I^-formyl-β-CD	6^I^-(*p*-toluenesulfonyl)-β-CD, DMSO, collidine, oil bath, 135 °C [47]	1.5 h	64
6^I^-formyl-β-CD	6^I^-(*p*-toluenesulfonyl)-β-CD, DMSO, collidine, MW (110 W), 135 °C [21]	15 min	68
6^I^-azido-6^I^-deoxy-β-CD	6^I^-(*p*-toluenesulfonyl)-β-CD, NaN_3_, DMF, oil bath 60–65 °C [48]	24 h	88
6^I^-azido-6^I^-deoxy-β-CD	6^I^-(*p*-toluenesulfonyl)-β-CD, NaN_3_, DMF, MW (200 W) 85 °C [15]	2 min	75
6^I^-deoxy-6^I^-thio-β-CD	6^I^-(*p*-toluenesulfonyl)-β-CD, thiourea, MeOH/H_2_O then HCl, oil bath, under reflux [49]	18 h	50
6^I^-deoxy-6^I^-thio-β-CD	6^I^-(*p*-toluenesulfonyl)-β-CD, thiourea, DMF then NaOH, MW (100 W), 100 °C [21]	20 min	69

#### Preparation of persubstituted CD derivatives

Selective permodification refers to a complete derivatization of the hydroxy groups in one side of the CD. The selective full substitution on the primary rim is not a trivial task because of the increase of steric hindrance that makes the secondary face prone to an attack before the last primary hydroxy group was reacted [50–51].

**Scheme 9:**
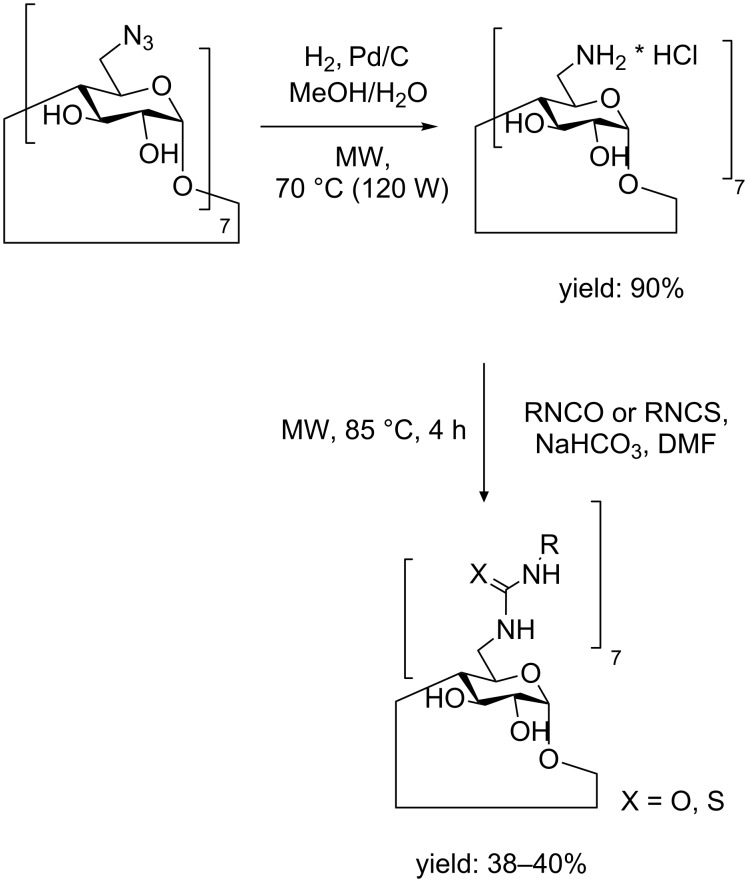
Synthetic scheme for the preparation of heptakis(6-amino-6-deoxy)-β-CD, heptakis(6-deoxy-6-ureido)-β-CD and heptakis(6-deoxy-6-thioureido-)-β-CD.

Pertosylate and perhalogenated derivatives in position 6 can be substituted with different nucleophiles. However, under conventional conditions, the reactions resulted in complicated mixtures with different substitution degree. MW irradiation efficiently afforded pure products. A series of amino derivatives were obtained by displacement of heptakis(6-deoxy-6-iodo)-β-CD (MW reactor 150 W) at 85 °C for 1 h (yield range 52–69%) [52]. Analogously catalytic hydrogenation in a pressure-resistant MW reactor, gave heptakis(6-amino-6-deoxy)-β-CD from a solution of heptakis(6-azido-6-deoxy)-β-CD in methanol/H_2_O [53]. The desired product was obtained in 90% yield after 3 h of irradiation at 70 °C. Reaction with isocyanates and isothiocyanate gave ureido and thioureido persubstituted β-CD derivatives in a MW oven at 85 °C for 4 h (see Scheme 9).

A multivalent azido-scaffold such as persubstituted 6-azido-6-deoxy-α-, β- or γ-CD with conformational constraints can be efficiently perfunctionalized in a MW- and ligand-assisted click cluster synthesis. An example of the MW-promoted ‘cooperative’ click reaction of azido-CDs has recently been reported and offers useful synthetic insights into a specific labelling strategy [54]. The aforementioned reaction afforded a new series of antimicrobial γ-CD derivatives that strongly disrupt bacterial membranes, and a series of persubstituted γ-CD derivatives bearing polyamino groups (77% yield) [55].

#### MW-promoted Cu-catalyzed click reaction for the preparation of second generation CD derivatives and hybrid structures

The MW-promoted CuAAC between CD monoazides and acetylenic moieties is the most efficient way to functionalize the CD surface [56]. β-CD is able to form a stable sandwich-type complex with Cu(II) ions, where the CDs faced their secondary rims and the use of heterogeneous phase catalysis may overcome the troubles deriving from time consuming purifications [57]. In 2006 Lipshutz et al. demonstrated that the impregnation of charcoal with an aqueous solution of Cu(NO_3_)_2_ in US bath, gave copper nanoparticles: an efficient catalyst in CuAAC [58]. Besides the easier work-up of heterogeneous catalysis, Cu(I)/charcoal also gave a higher yield compared to soluble CuSO_4_/ascorbic acid (76 vs 95% yield, respectively). The reaction was further improved under MW or simultaneous MW/US irradiation [59].

**Scheme 10:**
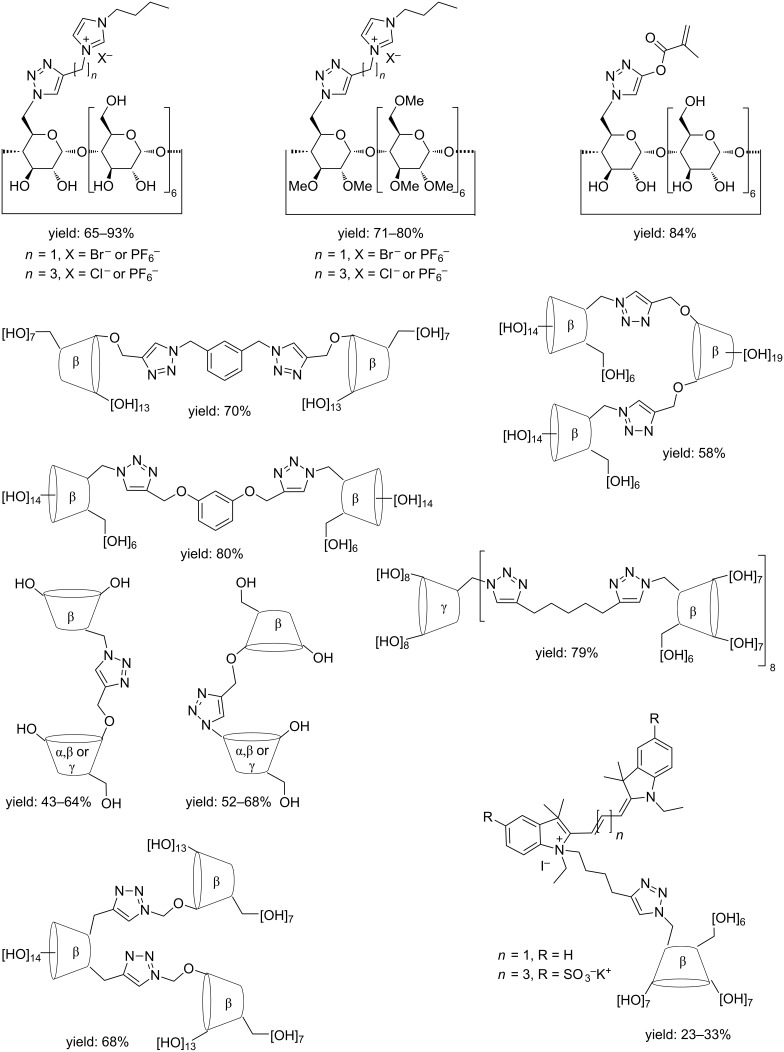
Structure of CD derivatives obtained via MW-assisted CuAAC.

Recently the preparation of a large number of CD-derivatives by MW-assisted CuAAC regioselective cycloadditions has been described. A selected series of derivatives are depicted in Scheme 10: CD-acryloyl derivative [60–61], β-CD/dye derivatives [31,62–64], CD-ionic liquid hybrids [65–66], CD-based iminosugar conjugates [67], water-soluble CD homo- and heterodimers [68–69], trimers [70–71] and oligomers [72] of α-, β- and γ-CD have all been successfully produced. This wide variety of compounds was obtained in good to excellent yield under MW irradiation (from 20 min to 3 h at 75 °C to 100 °C).

#### Preparation of CD-grafted materials and CD-based polymers

Interest in CD polymers has grown over the last few years. CD-based polymers have a number of applications, as drug delivery systems and toxic compounds scavengers, and have been obtained by grafting CDs into polymeric matrices.

**Scheme 11:**
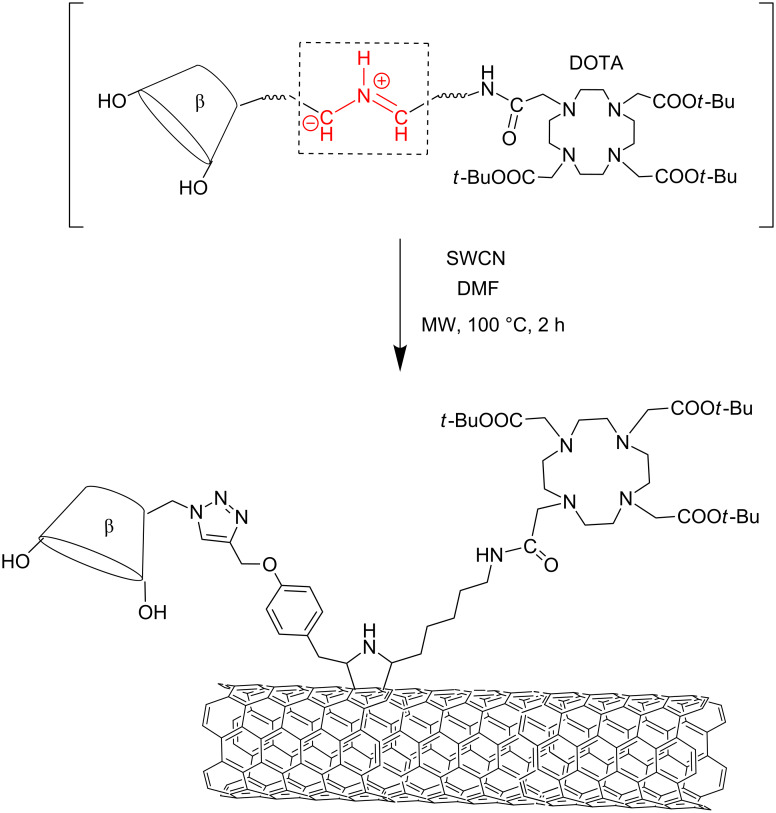
Preparation of SWCN CD-DOTA carrier.

A multi-carrier for combined diagnostic and theranostic applications was obtained via the functionalization of carbon nanotubes with CD using a MW-assisted 1,3-dipolar cycloaddition. As depicted in Scheme 11, the synthesis generated in situ azomethine ylides which include both a β-CD unit and a DOTAMA tris(*t*-butyl ester) moiety. The toxicity assessment, cell viability and permeability of single-walled carbon nanotube (SWCNT) platform, was evaluated on five human cell lines. No-toxicity was observed at concentrations up to 333 μg/mL [73].

Separately, a facile and rapid MW-assisted method in water has been used to derivatize graphene nanosheets with (2-hydroxy)propyl-β-CD. The reaction involved the esterification of the HP-β-CD hydroxy groups by the carboxyl groups of graphene oxide (GO) by MW irradiation (450 W) at different temperatures ranging from 50 to 100 °C for 10, 30, 60 and 90 min. After reduction with hydrazine hydrate, this HP-β-CD-RGO modified glassy carbon electrode showed good results in supramolecular recognition a set of six different phenolic organic pollutants and a high electrochemical response [74].

CuAAC has been successfully used to immobilize molecules on polymers and biopolymers as well as to join sugars to peptides and proteins. CD-polyglycerol dendron amphiphiles (CD-PG) have also been obtained. This derivative showed high encapsulation efficiency, while nanostructure size and shape were regulated according to the structure of the CD-PG dendrons [75].

CD-based polymers can be easily prepared under MW. Biswas et al. have prepared a number of macromolecular structures from α-, β-, γ-CDs by crosslinking reactions with toluene diisocyanate and methanediphenyl diisocyanate [76]. The authors demonstrated that compared with conventional heating, the reaction was faster (3–10 min) and with higher yields. Analogously, β-CD was grafted onto PEGylated Merrifield resin by reaction with HDI under MW irradiation [77].

CD nanosponges from anhydrous β-CD and diphenylcarbonate in DMF, have been prepared under MW irradiation (400 W) in 90 min. The optimized method was proven to be a unique opportunity for the large-scale synthesis of CD nanosponges in a high yield and uniform particle size distribution [78].

### Ball mill

One of the oldest, cheap, and efficient methods to achieve a homogeneous solid mixture is ball milling. By this method extremely fine powders can be achieved in mineral dressing processes, paints and pyrotechnics, etc. [79]. It is suitable for both batch and continuous operation, it is similarly suitable also for open and closed circuit grinding as well as being applicable for materials of all degrees of hardness.

Conventional BMs have a cylindrical or conical shell that rotates on a horizontal axis and have an appropriate grinding medium of balls, for example steel, flint or porcelain. The second generation of BMs, which are often called as high-speed ball mills (HSBM), operate in vibrating, mixer or planetary mode. A very simple vibrating BM, consisting of a small milling cup with one or two balls, has been used for a long time in traditional IR spectrometry to homogenize the sample and KBr. Mixer BM are slightly different from the vibrating version and are not only used in IR spectroscopy but also on the preparative scale for homogenization and cracking solid components. The common weakness of these simple accessories is the critical rotation/mixing speed, which can be overcome by a new generation of equipment; planetary BM, that consist of at least one grinding jar arranged eccentrically on a rotating support. The grinding jar moves in the opposite direction to the sun wheel. The difference in speeds between the balls and grinding jars produces an interaction between frictional and impact forces, which releases high dynamic energies for particles size reduction [80]. Detailed descriptions of both operating modes and theoretical considerations can be found and thoroughly discussed in various product brochures.

An energy efficient method for the preparation of nanocrystalline powders is the high energy ball milling (HEBM) in planetary or vibratory ball mills and HEBM is a common synonym for HSBM [81]. The lower particle size in grinding produces microdeformation in the ground material crystal lattice, while energy is partially spent in creating microstresses, which eventually slow powder grinding. An efficient wet grinding technology can exploit a liquid milling medium.

The preparation of CD and other complexes with the aid of ball milling is well-known [82–83]. In spite of an easy scale-up of this technology, some disadvantages might occur:

- metastable crystalline complexes can recrystallize to an equilibrium state upon storage [84];
- the degradation of mill surfaces and subsequent suspension contamination can be a problem, particularly in the high-energy version [85].

Although, the preparation of complexes or microparticles with ball milling is a common procedure, its use in organic synthesis intensified substantially only recently [86]. Solventless mechanochemical reactions are usually highly efficient and selective, valuable properties exploitable in CD derivatization.

Nucleophilic substitutions (S_N_2 reaction) may occur without solvent stabilization because charged species do not need to be formed in the transition state [87]. Solvent effects and ion pair formation are critical to the mechanism of S_N_1 reactions meaning that this mechanism is usually restricted in HSBM reactions.

While solid-state intermolecular S_N_2 reactions depend on contact between interacting particles only, S_N_1 reactions may show more structure-dependent behaviour, which can be either favourable or unfavourable, because of the solid-state structure.

Although BM reactions are often said to be solvent-free, some inert solvents can also be used particularly when the reagent mass ratio is very high. A lack of solvent(s) may suggest that ball milling conditions favours S_N_2 reactions; however it is also true that a solventless environment does not necessarily mean that there is a lack of solution in a liquid phase. Some reaction mixture components can often be liquid, while solvent effects or mixed S_N_2 and S_N_1-type reaction mechanisms cannot be excluded. A good example of a mixed reaction mechanism is the glycosylation reported by Tyagi et al. [88], where S_N_2 glycosylation seems to be dominant, with no neighbouring group participation, which is typical of glycosylation reactions of activated acetylated carbohydrates. A more pure S_N_2 reaction is described by Patil and Kartha [89], where the preparation of thioglycosides was almost quantitative. Unfortunately, a lack of information on reaction mixture compositions means that the reaction mechanism cannot be completely confirmed because chromatographic purifications and recrystallizations distort the enantiomeric ratio.

Basically, three major types of HSBM chemical reaction can occur in the presence of CDs:

1. Preparation of CD complexes and various chemical reactions on the complexed substructures;
2. Derivatization of naked, natural CD;
3. Reactions of activated CD.

While reactions occur between a complexed molecule and reagent or between host and guest in cases 1) and, usually, 2), reaction type 3) requires a CD derivative that bears a good leaving group and the complexation phenomenon can be disadvantageous here. While type 1) can eliminate usually the less problematic solvents only, the application of BM in types 2) and 3) can reduce or eliminate the polluting environment. Reactions of type 1) are dominant in CD/BM literature; more than 98% of publications report the complexation of one or more components. Mechanochemistry opened a new synthetic pathway to the preparation of numerous fullerene derivatives by dissolving C_60_ in the amorphous powder obtained from the ball milled reactants and β-CD [90]. Another example that uses the energy transfer of ball milling is the preparation of MnBi/Fe-Co core/shell structured composites. However, no pure chemical reaction is used to prepare rare-earth free ferromagnetic materials by grinding under less-environmentally friendly conditions in this case. The components were prepared using classic methods and the final composite was obtained by ball milling of arc-melted MnBi particles and Fe-Co nanoparticles prepared with the aid of a β-CD/oleic acid complex. The composites obtained showed smooth magnetic hysteresis loops [91].

SWCNT edge activation can be carried out via co-grinding with β- or γ-CDs [92]. Although chemical bonds are also broken in this case, this preparation is closer to the BM assisted preparation of CD complexes in many ways. Nanosized manganese oxides have also been prepared from CD/Mn complexes [93], however, in this case, the CD was only used to obtain a charrable matrix for the Mn_2_O_3_ which was prepared finally at 450 °C.

The only example of the type 2) method is the regioselective CD derivatization described by Menuel et al. who prepared 2-O-monotosylated α-, β-, and γ-CDs [94]. The further reaction of the prepared compounds resulted in a CD derived cyclic oligosaccharide, which contained one mannose residue, in the form of 2,3-mannoepoxide.

Type 3) reactions in the further derivatization of regioselectively activated – by sulfonic esters or halogenides – CDs are more important in industrial processes involving important CD derivatives. These activated derivatives are usually less soluble in water and their substitution reactions often require high boiling point dipolar aprotic solvents. The complete removal of these solvents is impossible even in gram scale preparations and so the prepared compounds need further purification steps. Additionally, these environmentally unfavorable solvents present other disadvantages; both in their decomposition and toxicology profile. A study of the nucleophilic displacement of 6-monosubstituted β-CDs and the synthesis of 6^I^-monoazido-6^I^-monodeoxy-β-CD in HSBM on a preparative scale (5 mmol, 6.5 g) is described in a recent publication by Jicsinszky et al. [95]*.* Comparing the yields it can be concluded that in larger scale reactions the yields are getting closer to those of the solution reaction. However, since the removal of a high-boiling solvent is not necessary, the work-up becomes simplifyed.

It has to be highlighted that the reaction product should not be considered as a CD derivative when the reaction centre is on the secondary rim because the S_N_1 mechanism is restricted to solution environment only. The secondary carbon substitution results in inversion in the reaction centre which changes the sugar moiety from glucoside to mannoside, altroside or alloside making those derivatives CD*-*based cyclic oligosaccharides and not CDs.

The design of green synthetic methods for the bulk preparation of CD thiols and thioethers is an emerging challenge because of the importance of intermediary azido derivatives [96] and favorable aggregation properties in nanomedicines and particularly the antidote Sugammadex [97]. The reaction between 6^I^-O-monotosyl-β-CD and various nucleophiles opens a new way for the more effective syntheses of per-6-substituted CDs from per-6-bromo- and -iodo-CDs.

### Microreactors

The typical lateral dimensions of microreactors, sometimes also called as microstructured or microchannel reactors, are below 1 mm with the most typical form of microchannels [98]. The miniaturized continuous flow reactor, also called microreactor, offers many advantages over conventional scale reactors, including considerable improved energy exploitation, increased reaction speed and yield, safety, reliability, scalability, on-site/on-demand production, etc., and a much finer degree of process control. However microreactors do not tolerate mechanical inhomogeneities. To resolve the problem of microparticles, which often cause clogging, a second generation of microreactors has been developed and called microjetreactor [99]. A typical microreactor is made up of a 'sandwich' of thin metal sheet or plates with fluid (micro)channels that have been etched into both sides. The average size of a single unit is approximately 6 × 4 × 0.5 cm with channel widths and wall thicknesses of 200–300 µm. The reactions occur in every other layer and the other layers are used for heat-exchange fluid flows [100].

The major use of CDs in this equipment, and also in the selective complexation phenomenon, is rather analytical and CDs' principal role is detection only [101]. This has allowed DNA sequencing to become a relatively cheap method and provided momentum to the discovery of the role of genetics in various diseases [102–103]. Although these reactors exhibit an excellent energy and mass efficacy, their use in CD derivatization is just a curiosity. However, exhausting the complexation ability of various CD derivatives is advantageous in solubilization and stereoselective reactions. Delattre and Vijayalakshmi have pointed out the theoretical use of enzymes in the production of CDs or other cyclic oligosaccharides, like cyclofructan, rather than using a microreactor [104].

## Conclusion

Dynamic intrusion of the enabling technologies to the CD chemistry is inevitable and shows exponential growth. Although, approximately 10% of the recently published technical papers in the CD field are dealing with sustainable technologies, the number of publications containing information of comparisons with the classical methods is sporadic. Optimized MW-, US- and BM-assisted protocols are energetically more efficient than the classical synthetic methods because their excellent heat and mass transfer. In all cases the reactions are faster avoiding degradations that may occur during protracted heating and time-consuming purifications. Case by case the technique of choice depends from several factors: the solubility of the starting CD, the reaction mechanism, environmental concerns, and the reaction scale are only a part of all the information required to design successful preparations.
